# The dynamic influence of nutrition on prolonged cognitive healthspan across the life course: A perspective review

**DOI:** 10.1016/j.nsa.2024.104072

**Published:** 2024-05-06

**Authors:** Curie Kim, Natalia Schilder, Katie Adolphus, Alessandra Berry, Chiara Musillo, Louise Dye, Francesca Cirulli, Aniko Korosi, Sandrine Thuret

**Affiliations:** a Basic and Clinical Neuroscience, Institute of Psychiatry, Psychology and Neuroscience, King's College London, UK; b Swammerdam Institute for Life Sciences, Center for Neuroscience, University of Amsterdam, Netherlands; c School of Psychology, University of Leeds, UK; d Center for Behavioral Sciences and Mental Health, Istituto Superiore di Sanità, Italy; e Institute for Sustainable Food, University of Sheffield, UK

**Keywords:** Cognition, Neurodevelopment, Ageing, Nutrition, Diet, Neurodegeneration, Energy intake, Early life stress, Gestational obesity, Obesity, Edentulism, Oral health, Memory, Antioxidants, Omega-3

## Abstract

Cognitive function undergoes a dynamic trajectory across the lifespan, influenced by multifaceted mechanisms encompassing genetics, lifestyle, and environmental factors. This review explores the impact of nutrition, specifically energy and bioactive components, on cognitive health across different life stages. Nutrition plays a pivotal role, influencing cognitive development, brain function, and age-related changes. Understanding these connections offers insights into strategies for extending cognitive healthspan. The review synthesizes evidence highlighting the nuanced effects of nutrition on cognition throughout life. Notably, age-specific energy balance emerges as a crucial factor in maintaining cognitive healthspan. Different life stages exhibit distinct nutritional demands affecting cognitive function. Maternal nutrition impacts prenatal and childhood cognitive development, while heightened glucose demands in adolescence influence long-term cognitive health. Midlife witnesses hormonal changes and decreased brain plasticity, while old age demands strategies addressing chronic lifestyle factors and energy restriction. We conclude that it is crucial to recognize the diverse requirements and biological changes across the lifespan and that deeper mechanistic investigations are vital to tailor nutritional recommendations for optimal cognitive health in all populations across the lifespan.

## Highlights

• Dynamic impact of nutrition on cognition throughout life facilitates targeted interventions.
• Maintaining nuanced age-specific energy balance may prolong cognitive healthspan.
• Enhanced healthy neurodevelopment with micronutrient and bioactive supplementation.
• Practical strategies such as oral hygiene and energy restriction in old age.

## 1 Introduction

Cognitive function, an important indicator of quality of life, has a dynamic trajectory across the lifespan. It begins with rapid and steep improvements during childhood neurodevelopment, stabilisation and maintenance during adulthood to a steady decline due to neural atrophy and degeneration during ageing (Gilmore et al., 2018; Peters, 2006; Craik and Bialystok, 2006). There is accumulating evidence that this cognitive evolution throughout life is influenced by diverse mechanisms encompassing genetics, lifestyle (e.g., exercise and nutrition) and environmental factors (e.g., stress, socioeconomic and education status) (Mollon et al., 2018; Dominguez et al., 2021; Jefferson et al., 2011). Many of these factors are modifiable, giving rise to the possibility of increasing cognitive healthspan into old age. However, the translation of evidence into specific requirements and strategies at different life stages and the critical window to target for interventions aimed at prolonging optimal cognitive function is unknown.

In particular, the impact of nutrition-related variables on cognitive function has garnered increasing attention, with accumulating evidence highlighting their significance. Nutrition and dietary intake are a complex subject which constitutes a source of energy and building blocks in addition to possessing key bioactive properties which may influence the brain via several identified pathways and mechanisms (Murphy et al., 2014). Above all, energy intake and the bioactive properties of food have been recognised as key facets of nutrition that may have a significant impact on the brain and cognitive function (Mattson et al., 2017). Similar to cognitive function, energy and nutrient needs also fluctuate throughout life with higher intakes required during pregnancy and early childhood which decline during adulthood and ageing. For energy specifically, the requirements for optimal brain functioning peak during childhood for rapid neurodevelopment, followed by accumulating evidence that, in fact, restricting energy intake may be more beneficial in later life (Kuzawa et al., 2014; de Cabo and Mattson, 2019). Therefore, targeting nutritional guidance around the differential life stages presents itself as viable target for building and maintaining cognitive health throughout life.

Overall, understanding how cognitive function can be influenced across the life-course provides insight into the mechanisms behind learning and memory as well as potential strategies to extend healthspan. This perspective review will discuss how the impact of nutrition, specifically energy and bioactive components, on cognitive health has been modelled and investigated, highlighting the differential areas of focus throughout the lifespan. Providing evidence of cause-effect mechanisms in humans poses a significant challenge due to numerous confounding factors arising from various intervening variables, e.g., genetics and environment. However, animal models offer a more accessible approach to address these questions, enabling the differentiation of confounders present in human studies, which are mostly observational in nature. Therefore, this review focuses on combining epidemiological and clinical evidence with outcomes from preclinical studies. The overall aim is to untangle the confounding variables in human studies and identify potential mechanisms of interest, facilitating the development of targeted intervention strategies at sensitive time-windows throughout life.

### 1.1 Prenatal

Early life experiences can embed biological traces into an individual's physiology, setting the stage for either vulnerability or resilience toward mental disorders. It is now widely accepted that stressful conditions experienced by the mother during pregnancy have the potential to affect foetal development, leading to negative health outcomes later in life. Besides overt psychiatric conditions that affect approximately 10% of pregnant women worldwide, other environmental challenges including living in a low socio-economic status environment, exposure to infection or malnutrition can affect foetal developmental trajectories (Sosnowski et al., 2018; Molenaar et al., 2019; Musillo et al., 2022; Reemst et al., 2022a). Overnutrition and obesity during gestation are emerging as a serious public health concern. Several epidemiological studies indicate that children of obese mothers have a greater chance of showing behavioural problems or being diagnosed with a neurodevelopmental disorder. These include attention deficit hyperactivity disorder (ADHD), autism spectrum disorders (ASDs) or developmental delay (Cirulli et al., 2020; Godfrey et al., 2017; Sanchez et al., 2018).

In the context of childhood cognitive function specifically, a relationship with gestational weight has been well documented. Those who gain the recommended -or even marginally above-amount of gestational weight have been associated with higher offspring intelligence quotients (IQ) and educational attainment (Gage et al., 2013; Hinkle et al., 2016). By contrast, a very large recent meta-analysis of 13 cohort studies from the United States, Europe and China reported that gestational weight gain above recommendations may be associated with better IQ, but reduced cognitive skills in language and mathematics in the offspring (Martínez-Hortelano et al., 2020). However, a gestational weight gain too far beyond the recommendations may lead to detrimental effects. For example, studies on the UK Millennium cohort have provided evidence for a negative relationship between maternal BMI and children's general cognitive ability at seven years (Basatemur et al., 2013). Similarly, a cohort study of over 11,000 children revealed that children of those in the highest quartile of maternal late-pregnancy BMI (>32.2 kg/m^2^) were characterised by lower performance in a variety of cognitive tests including executive function, processing speed, memory and school examination scores (Oken et al., 2021). Similar effects have also been seen with offspring IQ (Pugh et al., 2015).

Maternal obesity is mainly modelled by feeding dams before and/or during gestation with a high-fat diet (HFD). Overall, results from these studies complement and strengthen clinical and epidemiological evidence, indicating that offspring of obese mothers are characterized by social impairments, anxiety, an altered response to stress, cognitive disability, and hyperactivity (Sullivan et al., 2015; Musillo et al., 2023a). One important question that has been asked through preclinical models is whether pre-pregnancy and maternal obesity exert the same effects on the offspring. This is an important point that can redirect prevention policies: it is much easier to address weight gain problems during pregnancy than act upon an obesity condition that might be acquired much before gestation. A recent preclinical study appears to support epidemiological evidence indicating that prenatal and pregnancy windows have independent programming effects on the offspring. Preconception exposure affects body composition and adiposity while gestation exposure affects metabolism and tissue immune cell phenotypes (Chang et al., 2019). Overall, the current evidence suggests that pre-pregnancy obesity, rather than weight gain during gestation, may be most harmful to the foetus.

A great effort is currently being invested to address the specific biological mechanisms underlying the negative effects of maternal obesity on offspring brain development. Being pregnant and obese at the same time may turn out to be an extremely stressful condition leading to a maternal allostatic load that will engage multiple, not mutually exclusive, mechanisms during sensitive developmental phases. This may affect tissue organisation and organ physiology in the offspring. Among these mechanisms, hyperactivity of the HPA axis and the associated excessive glucocorticoids secretion as well as increased levels of oxidative stress and inflammation have been proposed to all contribute to mediate the effects of maternal obesity, ultimately increasing vulnerability to neurodevelopmental and psychiatric morbidity (Musillo et al., 2023a; Harris and Seckl, 2011; Bilbo and Tsang, 2010; Bordeleau et al., 2020; Volqvartz et al., 2023).

To address the need for feasible interventions, nutritional supplementation with antioxidant or anti-inflammatory agents are emerging as a promising strategy to prevent or counteract the detrimental effects of maternal obesity (Cirulli et al., 2022). In this context, the effects of the powerful antioxidant N-acetyl-cysteine (NAC) in a mouse model of maternal obesity have been widely characterised. Musillo et al. (2023) have recently provided evidence for prenatal NAC administration to be effective in counteracting the reduction in trophic factors (e.g., BDNF) and antioxidant defences (e.g., Nrf-2 and glutathione) in the offspring brain. Moreover, HPA axis functionality is rescued, and glucose homeostasis improved in a sex-dependent fashion in mice born to obese mothers (Musillo et al., 2023a; Berry et al., 2018). Overall, this evidence supports a key role of oxidative stress-related mechanisms in the long-term effects of maternal obesity and the effectiveness of a nutritional intervention strategy**.**

### 1.2 Childhood

Early life is a sensitive developmental period during which exposure to adverse events such as maternal depression, abuse, neglect, or malnutrition have a major impact on the development and function of the offspring's brain up till adulthood (Reemst et al., 2022b; Lucassen et al., 2013). Indeed, exposure to early life stress (ELS) prenatally and/or early postnatally is associated with an increased risk for mental and metabolic disease later in life (Reemst et al., 2022b).

Human evidence shows an association between ELS and adverse neurodevelopment and higher rates of mental illnesses including cognitive decline, Alzheimer's disease, anxiety, and depression in adulthood (Reemst et al., 2022b; Hoeijmakers et al., 2018; Yam et al., 2015). In recent years, our understanding of the potential underlying mechanisms contributing to these long-term effects of ELS has notably advanced. In humans, structural and functional changes in the brain following ELS have been identified (Yang et al., 2023). For example, studies have shown that ELS is associated with a reduction in gray matter volume and decreased hippocampal volume. Moreover, evidence shows functional and structural changes in cortical/limbic circuits, in particular the prefrontal cortex and the amygdala at different ages later in life (Yang et al., 2023).

The association between ELS and the brain has been substantiated with pre-clinical models (Hoeijmakers et al., 2018; Yam et al., 2015). A well-established preclinical model for ELS is based on introducing limited nesting and bedding (LNB) material from postnatal day 2–9 (Rice et al., 2008; Walker et al., 2017). Exposing mice to LNB has been shown to consistently lead to cognitive deficits and alterations in brain plasticity later in life (Naninck et al., 2015; Hoeijmakers et al., 2016; Abbink et al., 2019; Reemst et al., 2022c; Kotah et al., 2023). To be specific, LNB mice present with reduced hippocampal volume (Naninck et al., 2015) alongside alterations in microglial, astrocyte function and adult neurogenesis which might be key neurobiological substrates for the ELS-induced cognitive decline (Naninck et al., 2015; Hoeijmakers et al., 2016; Abbink et al., 2019; Reemst et al., 2022c).

Early-life adversity can take many different forms, both psychological and nutritional in nature. For example, malnutrition has been repeatedly shown to have profound effects on cognitive development of young children. A prospective, longitudinal study of 1559 children in the island of Mauritius at three years old found that malnourished children had poorer verbal ability, global IQ, reading ability, spatial ability, reading ability and school performance by 11 years old compared to those with no malnutrition (Liu et al., 2003). Moreover, a 55-year longitudinal study, the Barbados Nutrition Study, presented evidence that adults, previously malnourished during childhood, had impaired cognitive function, particularly attention and executive function (Roger et al., 2022). On the other side of the spectrum, a cross-sectional study of 233 children enrolled in the HOME study showed that increasing weight-for-height in developing children was inversely association with IQ scores, working reasoning and perceptual reasoning (Suryawan et al., 2022). In fact, a large-scale systematic review of suboptimal nutritional states from undernutrition/weight to obesity during childhood (in the first 60 months of age) found a consistent association with cognitive development in children in later life, including general cognition, attention, language and communication skills, mathematics and even motor development (Suryawan et al., 2022).

Nutritional status during early life also has a significant impact in adulthood. Later-life mental and metabolic diseases are often comorbid (Reemst et al., 2022b; Milaneschi et al., 2019a). Co-occurrence of obesity or diabetes with depression, cognitive decline or Alzheimer's disease have been described extensively (Reemst et al., 2022b), suggesting possible converging biological pathways (Milaneschi et al., 2019a). There is clinical and pre-clinical evidence that such comorbidity might have a common early origin. In fact, alongside an increased mental disease risk, exposure to ELS also increases the risk to metabolic and cardiovascular diseases including obesity (Reemst et al., 2022b). Not only does ELS lead to cognitive decline but also to long-term effects on the adipose tissue and leptin system, i.e., key hormones regulating appetite and fat storage (Yam et al., 2017a, 2017b). During the postnatal phase, ELS lead to a decreased body weight, reduced white adipose tissue, and altered fat composition with evidence of fat browning, suggesting modifications in thermogenesis. Such reduced fat mass persisted into adulthood. However, even though ELS mice exhibit an initially leaner phenotype, they showed increased fat accumulation upon exposure to a Western Style Diet (WSD). These results suggest that, in line with the human evidence of ELS leading to increased vulnerability for metabolic derangements, ELS exposure leads to higher vulnerability to develop obesity in such moderate obesogenic environment.

Although the exact mechanisms of this ELS-induced metabolic vulnerability are largely unknown, the hypothalamus plays a central role in this observed interplay. The hypothalamic circuity develops early in life and has a central role in food related decision making, under control of the metabolic hormones, ghrelin, insulin and leptin (Yam et al., 2017a). Strikingly, ELS has been shown to disrupt developmental ghrelin and insulin levels in a sex- and age-specific manner as well as impact hypothalamic fibre density of key neuropeptides involved in feeding circuits (Yam et al., 2017a).

Considering the strong interrelation between stress and nutrition, along with high comorbidity of mental and metabolic derangements, their converging biological substrates, and their common early-life origin, peripheral or systemic interventions emerge as a promising approach. These interventions aim to protect against ELS-induced effects. Nutritional strategies have recently received increasing attention in modulating mental and metabolic health (Marx et al., 2021; Adan et al., 2019) and early life nutrition, in particular, might be key (Juncker et al., 2022). Early life nutrition is a crucial aspect for brain development since it is the fastest growing organ with high metabolic activity and energy demand (Lucassen et al., 2013). In fact, minor nutritional deficiencies can have far reaching effects on brain structure and functioning (Juncker et al., 2022). This makes early life diet a prominent candidate in modulating the described ELS-induced effects.

Early nutritional interventions have been shown to effectively protect against the lasting ELS-induced effects on cognition. Early supplementation with essential micronutrients choline, folic acid, methionine, zinc, and vitamins B6 and B12, given from postnatal day 2 till postnatal day 9 restored ELS-induced (via LBN from P2–P9 exposure) depletion of brain and plasma methionine and rescued adult cognitive impairments as confirmed by hippocampus-dependent tasks (Naninck et al., 2017). These effects appeared to be mediated, at least partly, by the mitigation by the diet of the ELS-induced hypothalamus pituitary adrenal axis hyperactivity. Furthermore, an early diet which increased the availability of omega 3 long-chain polyunsaturated fatty acids (PUFAs – i.e. low in the ratio of linoleic acid (LA, Ω-6)/α-linolenic acid (ALA, Ω-3)) provided from postnatal day 2 until postnatal day 42 has been shown to prevent ELS-induced adulthood cognitive impairments and the associated reduction of hippocampal cell survival and increase in phagocytic microglia (Reemst et al., 2022c; Yam et al., 2019). In addition, early dietary FA ratio was shown to alter the brain lipidome and oxylipins (PUFA derivatives) profiles potentially contributing to the beneficial effect of the diet (Reemst et al., 2022c).

These studies provide an evidence base for the effectiveness of early nutritional strategies to protect against cognitive decline in vulnerable populations exposed to ELS modulating. An improved mechanistic understanding of the interplay between early life stress, nutrition, mental and metabolic health opens incredible opportunities for research directed towards future nutrition-based clinical applications.

### 1.3 Adolescence

As we move further into adolescence, most studies have considered the impact of breakfast on cognitive function. This may be due to the high prevalence of breakfast skipping during adolescence (Lazzeri et al., 2023). Previous studies on breakfast and cognitive function have considered the acute effects of a single breakfast meal that occur shortly after breakfast consumption on the same morning of consumption following an overnight fast (Adolphus et al., 2016). The evidence from acute intervention studies comparing breakfast versus (vs.) no breakfast suggest that consuming breakfast has an acute beneficial effect on cognitive function measured within 4 h post-ingestion in adolescents (Cooper et al., 2011; Defeyter and Russo, 2013; Widenhorn-Müller et al., 2008; Wesnes et al., 2003; Adolphus et al., 2021). The positive effects appear to be domain-specific, such that tasks requiring attention, executive function, and memory are facilitated more reliably by breakfast consumption relative to fasting.

Several studies have considered the effects of breakfast on academic performance in terms of school grades and achievement test scores, which reflect typical performance indicators used within the education system. This evidence demonstrates that habitual breakfast consumption frequency is positively associated with academic performance in adolescents (Boschloo et al., 2012; Lien, 2007; Øverby et al., 2013; So, 2012). Most support a positive effect in terms of improvements on academic performance outcomes particularly mathematics and arithmetic attainment. A recent cross-sectional study demonstrated that habitual school-day breakfast consumption frequency was positively associated with academic performance, as measured by the General Certificate of Secondary Education (GCSE), which is a national academic qualification obtained by most British adolescents during secondary education (Adolphus et al., 2019). Those who rarely ate breakfast scored on average 10.25 points lower than those who frequently ate breakfast, a difference of nearly two grades, after accounting for other important factors including socio-economic status, ethnicity, age, sex, and BMI (Adolphus et al., 2019).

Certain micronutrients, including iron and iodine, are associated with cognition in adolescents. This is important given that inadequate iron and iodine intake is common among adolescents (Bath et al., 2022; Ferrari et al., 2011). A recent systematic review and meta-analysis demonstrated that iron supplementation has a significant positive effect on the intelligence, attention, and memory of school-age children, including adolescents, but there was no evidence on the effect of iron supplementation on school achievement (Gutema et al., 2023). Moreover, micronutrient supplementation has been shown to have a positive effect on fluid intelligence among micronutrient-deficient school-age children (4–18 years), especially those who were iron-deficient or iodine-deficient at baseline (Lam and Lawlis, 2017). There is also evidence that mild gestational iodine deficiency results in adverse neurocognitive impacts on offspring. A 15- year follow up of the Gestational Iodine Cohort demonstrated that reduced literacy and numeracy outcomes persist into adolescence following mild iodine deficiency in utero, despite growing-up in an iodine replete environment in childhood (Hynes et al., 2017).

### 1.4 Midlife

Midlife is a crucial time bridging growth and decline, youth and old age (Lachman et al., 2015). Characterised by changes in metabolic balance, hormonal secretion, brain and behavioural plasticity as well as by a decreased ability to cope with stress, middle age can confer vulnerability to the onset or progression of physical and mental health issues. Cognitive decline is a prevalent condition among the elderly which has significant impact on quality of life during aging, ultimately decreasing healthspan.

A clear association between midlife obesity and cognitive decline has been reported (Hartanto et al., 2019; Dye et al., 2017). In particular, midlife obesity is a significant risk factor for the development of Alzheimer's Disease (AD) and other forms of dementia (Xu et al., 2011; Dahl et al., 2013). Moreover, evidence suggests that excessive overweight at this age may have an immediate detrimental impact on cognitive functions, particularly those related to memory (Koutsonida et al., 2023). For example, poor behavioural flexibility and decision-making have been reported in obese people. This behaviour could underlie the preference for immediate high-calorie palatable food rewards and provide an explanatory mechanism for control of energy intake which plays a pivotal role for health outcomes (Dye et al., 2017). Notwithstanding this evidence, data on the relationship between cognitive abilities and obesity are conflicting with confounds such as obesity-associated comorbidities including hypertension, hypercholesterolaemia, insulin resistance (IR) and type-2-diabetes (T2DM) that often co-exist in obese individuals (Gunstad et al., 2006; Cournot et al., 2006; Cheke et al., 2016; Gonzales et al., 2010; Nilsson and Nilsson, 2009; Eichen et al., 2023). In fact, studies have shown that impaired glucose tolerance *per se*, is sufficient to negatively affect performance of hippocampal-dependent cognitive tasks. This suggests that elevated glucose levels may also increase the risk of cognitive dysfunction in non-diabetic individuals (Lamport et al., 2009; Crane et al., 2013; Anstey et al., 2015).

During midlife, the functioning of the hypothalamic-pituitary-adrenal (HPA) axis undergoes physiological changes. These changes, along with shifts in the circadian rhythm of glucocorticoid secretion, may impact stress responsiveness and the ability to cope with everyday challenges. Many psychiatric conditions are co-morbid with metabolic dysfunctions suggesting that the neuroendocrine and energy homeostatic systems share several signalling pathways that may be modulated by stress (Musillo et al., 2022; Reemst et al., 2022a; Cirulli and Alleva, 2009; Milaneschi et al., 2019b). For example, Brain-Derived Neurotrophic Factor (BDNF), a neurotrophin which plays a role in brain plasticity, cognitive processes, and emotion regulation, is highly expressed in both the hypothalamus and hippocampus, two brain regions playing key roles in energy balance, stress responsiveness and cognitive functions (Cirulli and Alleva, 2009; Cirulli et al., 2004). Moreover, acute stress can promote the consumption of highly sweetened comfort food or trigger behaviours such as binge eating that may help the individual to cope with unpleasant emotions by blunting HPA axis activation (Ulrich-Lai et al., 2011). However, in the longer term, this compensatory mechanism leads to excessive visceral fat accumulation and the onset of obesity-related disorders (eg. metabolic syndrome) that have been associated with increased oxidative stress, chronic inflammation, and impaired cognitive performance, overall resembling a form of precocious (brain) aging (Tchkonia et al., 2010; Armani et al., 2017).

The brain is particularly vulnerable to oxidative stress insults since it is characterized by a high metabolic rate, poor antioxidant defences and low ability for cellular regeneration (Finkel and Holbrook, 2000). However, reactive oxygen species (ROS), that play a key role in brain aging, are not only a by-product of metabolic processes responsible for direct oxidative damage, but they may also be involved in specific signalling pathways. A striking example is hydrogen peroxide (H_2_O_2_) which by specifically reinforcing insulin signalling within the adipocyte, may promote fat accumulation. Pivotal to this process is the p66Shc *gerontogene* which increases the generation of H_2_O_2_, amplifying insulin signalling which ultimately leads to impaired β-cell function and insulin resistance as a result of excessive body fat accumulation (Berniakovich et al., 2008; Trinei et al., 2009). Interestingly, deletion of p66Shc gene in mice resulted in reduced levels of inflammation and oxidative stress. These features are associated with reduced fat accumulation and improved cognitive abilities, strengthening the link among metabolism, oxidative stress, brain function and behaviour (Biondi et al., 2022). Consequently, challenges to this balance, amplified by aging progression, may profoundly affect health outcomes paving the way for cognitive decline and dementia later in life.

### 1.5 Old age

The United Nations Department of Economic and Social Affairs have reported that between 2015 and 2030 the population of individuals aged 60 and over is expected to grow by 56% to more than 1.4 billion i.e., 1 in 6 people globally (P D United Nations Department of Economic and Social Affairs, 2015). Since 1999 Europe already has seen a shift in population demographics in which there are more people over the age of 60 than under 15 years old (Eurostat, 2021). One of the most rapidly increasing health conditions directly related to the growing ageing population is dementia, a leading contributor to disability. The number of individuals suffering from dementia is projected to double every 20 years to 115.4 million globally by 2050 (Prince et al., 2013).

In older populations the maintenance and rescue of cognitive function is a crucial target, as opposed to the development that is prioritised in earlier years. As such, there is a focus on uncovering practical interventions and strategies that could be employed in later life. Similar to adolescence, the importance of maintaining glucose consumption for healthy brain function is also critical maintaining cognitive healthspan throughout age. Ageing-related neurodegenerative conditions such as dementia and Alzheimer's disease are accompanied by impaired brain glucose metabolism function (Xu et al., 2023; Cunnane et al., 2020). As a response, therapeutic interventions using ketone bodies, an alternative fuel source to glucose, are currently being investigated with promising results. For example, a ketogenic drink has been shown to improve brain energy status and improve several cognitive measures, including episodic memory, in those with mild cognitive impairment (MCI) (Fortier et al., 2019). Similarly, six weeks of carbohydrate restriction to induce ketone metabolism was shown to improve memory performance in MCI (Krikorian et al., 2012). Other interventions that are of interest to restore brain glucose metabolism include increasing insulin sensitivity, antioxidant supplementation, and reversing mitochondrial dysfunction – albeit predominantly in animal models (Cunnane et al., 2020).

Another widely investigated intervention is energy restriction (ER), whereby energy intake is reduced with the absence of malnutrition. ER has been robustly shown to increase lifespan and decrease ageing-related diseases in animal models, including rodents, flies, and non-human primates (Fontana and Partridge, 2015). Several mechanisms by which ER might exert a positive influence on lifespan have been explored in animal and human studies. These include reduced oxidative damage, upregulation of growth factors e.g., insulin growth factor 1, and changes in energy metabolism (Sohal and Weindruch, 1979; Fontana et al., 2010; Anderson et al., 2009). In the context of cognition, ER has been shown to improve cognitive function in animal models, in particular hippocampus-dependent learning and memory through mechanisms such as increased adult hippocampal neurogenesis (Park et al., 2013; Hornsby et al., 2016; Lee et al., 2002). Moreover, in human studies, ER has been associated with improvements in risk factors for cognitive impairment such as cardiometabolic health, reduced oxidative stress and inflammation, and memory function including hippocampus-dependent cognition (Huffman et al., 2022; Redman et al., 2018; Kim et al., 2020; Witte et al., 2009).

An important and lesser considered factor which contributes to energy intake, particularly in older populations, is oral health, which becomes a more prominent issue in ageing. Old age is accompanied by worsening oral health, such as increased periodontal disease and edentulism (Thomson and Ma, 2014). In the context of energy intake, this is an interesting angle to consider when discussing strategies to prevent or slow down ageing-related cognitive decline. Softer foods, which are easier to consume when an individual suffers from problems with oral health and edentulism, have been associated with increased energy intake compared to harder foods. For example, Bolhuis et al. (2014) demonstrated that harder foods consumed at lunch time led to a 13% lower energy intake compared to soft food with no impact on fullness and energy intake later in the day at dinner. Moreover, an association has been shown between a lower chew rate (chews per minute) and obesity (Borvornparadorn et al., 2019).

Although texture modification of food is a viable way to ensure adequate nutrient intake in ageing populations which have higher rates of dysphagia (Raheem et al., 2021), a positive effect of mastication on human brain function has emerged. For example, a chronic 3-month chewing intervention in habitual non-chewers has been shown to improve hippocampus-dependent recognition memory (Kim et al., 2019). In support, the ability to chew has been associated with superior function in several cognitive domains including global intellectual function and verbal memory (Moriya et al., 2011; Kimura et al., 2013; Weijenberg et al., 2015; Lee et al., 2013). These associations have been replicated by cohorts from many different countries and research groups (Naorungroj et al., 2015; Okamoto et al., 2015; Tsakos et al., 2015).

The link between oral health, edentulism and cognitive decline is clearly shown by the extensive amount of evidence discussed above. A relationship with pathological signs of neurodegeneration is less known. However, since the 90s there is a growing body of evidence suggesting that the higher numbers of teeth in elderly populations is associated with mild cognitive impairment and dementia, some reporting up to 91% increased risk (Kondo et al., 1994; Luo et al., 2015; Choi et al., 2022; Lu et al., 2021; Jones et al., 2023). Despite the evidence, it is still unclear how oral health and cognitive status are related. There is no overall consensus that has been reached between studies, likely due to inconsistent cognitive testing and oral assessment (Wu et al., 2016). Moreover, it is important to consider reverse causality. The poor oral health and increasing numbers of missing teeth in those with dementia may be due to the inability to upkeep oral hygiene practices as a result of cognitive impairment (Moritz et al., 1995).

## 2 Conclusion

**Fig. 1:**
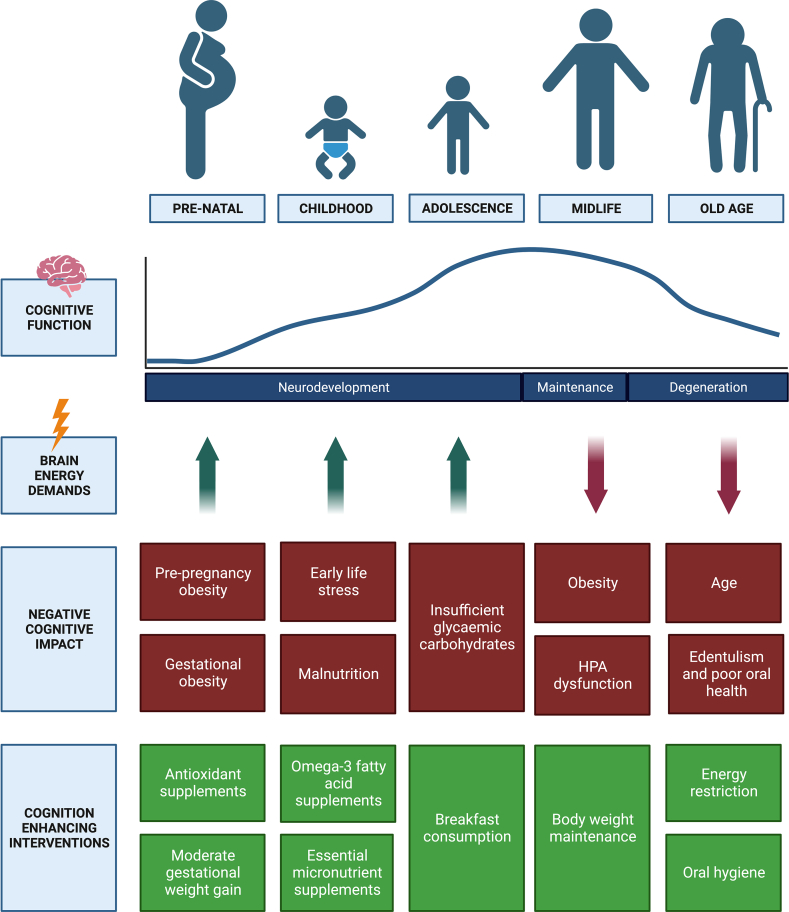
**The dynamic trajectory of cognitive function and nutritional demands throughout life.** Cognitive function improves rapidly during childhood neurodevelopment, followed by stabilisation and maintenance during adulthood to a steady decline during ageing. This pattern aligns with the fluctuating energy and nutritional demands of the brain at each life stage, presenting opportunities for targeted nutritional interventions. These interventions have the potential to enhance and prolong cognitive healthspan. HPA: Hypothalamic-pituitary axis. Figure created with BioRender.com.

Age-specific nutritional demands are an important regulator of cognitive function, largely by preventing cascade effects ranging from overnutrition, resulting in obesity or malnutrition, to increased inflammation and impaired stress responses. As discussed above, it appears reasonable to employ diverse strategies throughout the lifespan with each life stage characterised by distinct changes and associated targets (summarized in Fig. 1). In the pre-natal and childhood phase, the emphasis lies on high maternal BMI and nutrition-related ELS which impact cognitive development. However, the distinct feature in adolescence is heightened glucose demands of the brain which has been shown consistently to be a pivotal factor in healthy cognitive development. In response, nutritional supplementations are a promising avenue to protect against the lasting impact on later life cognitive function. Moving into midlife, an increased rate of ageing-related hormonal and metabolic changes coupled with decreased brain plasticity become prominent features. Lastly, in old age, the amalgamation of chronic lifestyle factors, such as oral hygiene practices, and the potential for energy restriction to prolong healthspan and lifespan allow for practical strategies to slow down cognitive abilities. Moreover, amongst all age groups a nuanced energy balance is critical in order to prevent harmful energy deficits through malnutrition and obesity as a result of overnutrition.

To effectively provide personalised advice and implement strategic policy making it is important to consider factors beyond just age. Vulnerable populations, such as those lacking food security, low socioeconomic status, or chronic disease must be considered and require nuanced interventions. In addition, investigating the diversity within studies is essential to provide culturally sensitive recommendations. Moreover, it has been shown in several large-scale studies that women are disproportionately affected by cognition-related diseases such as Alzheimer's disease (Laws et al., 2018). Therefore, future studies should consider sex differences, drawing insights from animal models, large cohorts and intervention studies.

Nutritional interventions represent a promising strategy to be employed throughout life, in some cases perhaps even preferable to classic medications leading to greater consumer acceptance and lower side effects. However, assess the efficacy of nutrients on cognition and brain health is very complex and require further efforts (Adan et al., 2022). Thus far, there are major gaps in our understanding at each life stage which are summarized in Table 1. The challenge of employing nutritional strategies for brain health goes beyond individual responsibility and requires a holistic approach in which governments, food industry, and society take a co-ordinated approach (Adan et al., 2022). As such, the policy implications (Table 1) of the evidence presented must also be carefully considered and applied effectively.

**Table 1:** A life-stage specific summary of the current gaps in our knowledge that must be addressed to improve upon the current policy implications in the field.

Life Stage	Major Knowledge Gaps	Policy Implications of Existing Evidence
Pre-natal	Nutrients act through multiple synergistic mechanisms, which are especially complicated during pregnancy, when any intervention simultaneously affects both the mother and the foetus (Merialdi et al., 2003). It is crucial to understand the interaction of internal factors, such as hormones, overall immune regulation, and metabolism with nutrition or food choices.	Since healthy brain function in adulthood is dependent on exposure to early dietary and lifestyle factors, society and practitioners must be aware that the prenatal and early postnatal period are fundamental to build foundations for prolonged healthspan. Education of beneficial nutritional practices should be highlighted and prioritised to the mother.
Childhood	Several psychopathologies present comorbidities with metabolic and cardiovascular diseases (Laws et al., 2018). Whether early-life adversity is the origin of such comorbidity, converging on cellular metabolic pathways in different tissues and key hormones that regulate appetite and fat storage remains to be investigated. One important connection between human cognition and metabolism is the gut-brain axis (Tooley, 2020). The gut microbiome is strongly age- and diet-dependent as well as highly sensitive to the environmental stimuli (e.g. stress) and might thus be an important player in cognitive and metabolic health. How early life adversity affect the gut-brain communication and if such alterations contribute to the long-term impact of early-life adversity on later life cognitive and metabolic health remains to be determined.	Nutritional interventions are a promising avenue to combat early-life adversity induced cognitive decline. It is key that nutritional strategies are being incorporated in training programs of medical specialists, the education system as well as communicated to policy makers.
Adolescence	The relationship between cognition and dietary habits have predominantly investigated in young children whilst adolescents are under researched. Adolescents present with increased breakfast skipping, greater agency over food intake it has been observed that habits formed in childhood and adolescence tend to continue into adult life (Sincovich et al., 2022). Therefore, it is crucial to focus attention on this particular age group in order to identify preventative strategies.	School breakfast programs have been shown to be cost-effective, improve school attendance and increase the likelihood of achieving recommended daily intakes of nutrients, such as iron (Bartfeld et al., 2019). In addition, programmes such as free dental care to form the foundations for long term healthy behaviours which may impact the onset of cognitive in later life would be beneficial.
Midlife	Supplementation via nutraceutical compounds, such as polyphenols which might counteract low-grade inflammation and oxidative stress with reduced side effects, is very promising. However, the mechanisms of action of polyphenols are multitarget and are overall still unclear. Moreover, their effects appear to be finely modulated according to gender differences (Musillo et al., 2023b), the mechanisms for which are not fully understood.	While future research should target middle-age as a time window of opportunity to efficiently prevent, rather than counteract, cognitive decline, joint efforts should be devoted to better clarify the efficacy of specific nutrients (including polyphenols) on cognition to meet the requirement of precision medicine.
Old Age	Ageing populations often present with several co-morbidities that contribute to impaired cognitive function, including, diabetes and cardiovascular disease (Samieri et al., 2018). Converging pathways between the impact of nutrition on these conditions and cognitive decline would be informative in streamlining treatments and is yet to be fully determined. In addition, it has been shown that there is a differing prevalence in Alzheimer's disease and many other age-related conditions dependent on sex. Therefore, it is vital to investigate sex-differences (Laws et al., 2018) in the context of nutritional interventions.	Practical interventions to address the maintenance and rescue of cognitive function should be prioritised alongside preventative practices. These interventions should be integrated into existing healthcare practices to minimise the continuing burden on healthcare systems by the increasing rate of age-related condition.

While it remains important to find a common pathway that can be attainably targeted, it is equally essential to recognize the diverse requirements and biological changes that occur throughout the lifespan. This variability calls for deeper investigations into differential mechanisms to effectively target nutritional recommendations that ensure optimal cognitive health throughout life in all populations.

## Funding

10.13039/501100014566FC was supported by ERANET NEURON JTC 2018 (Mental Disorders) Project ‘‘EMBED”.

CM was supported by “Avvio alla Ricerca - Tipo 1” 2021 “Sapienza” University of Rome funding to C.M.

AK was supported by 10.13039/501100010969Alzheimer Nederland, ZonMW and 10.13039/100013278JPND.

10.13039/501100001515CK was supported by the Reta Lila Weston Trust.

The above funding sources were not involved in the writing of the manuscript nor in the decision to submit the article for publication.

## Declaration of competing interest

KA is a current employee of Tate & Lyle PLC, UK.
